# Co-creation of activities to promote health and well-being of older people – a scoping review

**DOI:** 10.1093/eurpub/ckac129.279

**Published:** 2022-10-25

**Authors:** AS Terkelsen, CT Wester, G Gulis, J Jespersen, PT Andersen

**Affiliations:** 1 Public Health, University of Southern Denmark, Esbjerg, Denmark; 2 Public Health, University of Southern Denmark, Odense, Denmark

## Abstract

**Background:**

The global population is ageing and the need to promote health and well-being of this generation is essential. Co-creative practices can be solutions to welfare challenges in the health care sector and local policies. However, literature addressing co-creation of activities to promote health and well-being is sparse. The review aimed to identify health promotive activities co-created between the public and older people, the influence of co-creative activities on health and well-being of older people, and facilitators and barriers for doing co-creation.

**Methods:**

We searched for peer-reviewed and grey literature in eight scientific and five non-scientific databases. Two reviewers independently screened publications for eligibility according to inclusion and exclusion criteria and extracted data. An inductive thematic content analysis was applied for the analysis.

**Results:**

We included nineteen publications. Four themes related to co-creative activities emerged: “Social activities”, “Activities to create age-friendly environments”, “Discussions of healthy ageing”, and “Physical activities”. The co-creative activities influenced the overall well-being, and promoted active and healthy ageing, physical functioning, and quality of life. Identified facilitators for co-creation were the role of the facilitator, a supportive environment, recognition of competencies, while the main barriers were time and resources, and recruitment of participants.

**Conclusions:**

Few studies have investigated co-creation of activities to promote health and well-being of older people. The included studies dealt with activities in any form and not merely social and physical activities co-created. Future co-creation of activities with older people should consider the role of facilitators, the environment in which the co-creation takes place and value time, resources, and competencies of participants.

**Key messages:**

• Studies on co-creation of activities to promote health and well-being of older people is sparse and must be explored further.

• Future research may focus on co-creation of social and physical activities to promote health and well-being of older people and consider known facilitators for co-creation.

